# Patient preferences for emergency department-initiated tobacco interventions: a multicenter cross-sectional study of current smokers

**DOI:** 10.1186/1940-0640-7-4

**Published:** 2012-03-15

**Authors:** Esther K Choo, Ashley F Sullivan, Frank LoVecchio, John N Perret, Carlos A Camargo, Edwin D Boudreaux

**Affiliations:** 1 Injury Prevention Center, Department of Emergency Medicine, Rhode Island Hospital, Warren Alpert Medical School of Brown University, 55 Claverick Street, 2nd Floor, Providence, RI 02903, USA; 2 Emergency Medicine Network, Department of Emergency Medicine, Massachusetts General Hospital, 326 Cambridge Street, Suite 410, Boston, MA 02114, USA; 3 Department of Emergency Medicine, Maricopa Medical Center, 2601 East Roosevelt Street, Phoenix, AZ 85006, USA; 4 Department of Emergency Medicine, LSU Health Sciences Center/Earl K. Long Medical Center, 5825 Airline Highway, Baton Rouge, LA 70805, USA; 5 Emergency Medicine Network, Department of Emergency Medicine, Massachusetts General Hospital, 326 Cambridge Street, Suite 410, Boston, MA 02114, USA

**Keywords:** Smoking, Tobacco, Cigarettes, Emergency medicine, Counseling, Patient preference

## Abstract

**Background:**

The emergency department (ED) visit provides a great opportunity to initiate interventions for smoking cessation. However, little is known about ED patient preferences for receiving smoking cessation interventions or correlates of interest in tobacco counseling.

**Methods:**

ED patients at 10 US medical centers were surveyed about preferences for hypothetical smoking cessation interventions and specific counseling styles. Multivariable linear regression determined correlates of receptivity to bedside counseling.

**Results:**

Three hundred seventy-five patients were enrolled; 46% smoked at least one pack of cigarettes per day, and 11% had a smoking-related diagnosis. Most participants (75%) reported interest in at least one intervention. Medications were the most popular (e.g., nicotine replacement therapy, 54%), followed by linkages to hotlines or other outpatient counseling (33-42%), then counseling during the ED visit (33%). Counseling styles rated most favorably involved individualized feedback (54%), avoidance skill-building (53%), and emphasis on autonomy (53%). In univariable analysis, age (r = 0.09), gender (average Likert score = 2.75 for men, 2.42 for women), education (average Likert score = 2.92 for non-high school graduates, 2.44 for high school graduates), and presence of smoking-related symptoms (r = 0.10) were significant at the *p *< 0.10 level and thus were retained for the final model. In multivariable linear regression, male gender, lower education, and smoking-related symptoms were independent correlates of increased receptivity to ED-based smoking counseling.

**Conclusions:**

In this multicenter study, smokers reported receptivity to ED-initiated interventions. However, there was variability in individual preferences for intervention type and counseling styles. To be effective in reducing smoking among its patients, the ED should offer a range of tobacco intervention options.

## Background

Tobacco remains the leading preventable cause of morbidity and mortality in the United States (US), increasing the risk of coronary artery disease, stroke, lung cancer, and chronic obstructive pulmonary disease and accounting for 443,000 deaths (nearly 1 in 5) each year [1]. Tobacco use and tobacco-related illnesses are common among emergency department (ED) patients. Studies performed in multiple ED settings have found that 30-40% of ED patients smoke--prevalence rates that are well above that of the general population [2,3].

Although providing smoking counseling has not traditionally been a function of emergency-care services, the field is increasingly recognizing the necessity and practicality of assuming such functions. As the number of primary care physicians declines [4], ED volume is steadily increasing, and health-care reform is not projected to significantly reduce this high utilization over the next decade. In 2006, there were 40.5 ED visits per 100 people in the US, an 18% increase from 1996 [5]. Unfortunately, many individuals do not have a stable medical home and fail to follow up with other care providers after the ED visit [6-8]. The ED remains an episodic but frequent point of health-care contact for much of the population, making the visit itself the ideal, and possibly only, opportunity to provide behavioral interventions.

Prior studies have found that adult smokers in the ED are motivated to quit [9], with 61-79% in the contemplation or preparation stage of change [10]. There is also evidence that smokers in the ED are interested in receiving a cessation intervention during their visit [10], and that smoking cessation interventions by ED physicians improve patient satisfaction with their visit [11]. However, much remains to be discovered about exactly what kinds of interventions are most feasible and effective during the ED visit [12]. Motivational interviewing (MI) has been used as the basis for alcohol and drug interventions in the ED [13-15], and the evidence for MI for smoking cessation in general is positive [16,17]. The US Preventive Services Task Force (USPSTF) recommends motivational interviewing as one of the "practices that improve cessation rates" [18]. However, the effectiveness of MI for smoking cessation in the ED setting, where interventions generally occur in a brief single session, is unknown. Nor is there evidence regarding other potential approaches to smoking cessation, including alternate types of in-ED counseling, use of referrals to more intensive outpatient counseling, nicotine replacement therapy (NRT), or other medical therapies. The lack of, and need for, current knowledge in ED-based brief interventions for tobacco use has been openly acknowledged within the field of emergency medicine [12].

Incorporating patient values and preferences is considered a key step in developing interventions and treatment guidelines in medicine [19,20]. Specifically, exploring patient attitudes toward the variety of potential interventions and services would allow health-care providers in the ED to focus their efforts on developing interventions that are more likely to gain traction in its patient population. Further, identifying preferences based on patient characteristics would allow interventions to be tailored to specific subgroups or individuals, if indicated. Understanding patient preferences could also allow the ED to identify discrepancies between what patients find acceptable and/or desirable and what ED clinicians and systems are currently prepared to provide.

This article reports our findings from a multicenter cross-sectional study of ED patients who reported current smoking. We describe ED patient preferences regarding the format, intensity, and style of specific potential tobacco interventions; the association between patient characteristics and interest in receiving ED-based smoking cessation counseling; and the smoking-related assessment and services actually provided, based on documentation by clinicians in the medical chart.

## Methods

### Study design

This was a cross-sectional study conducted at 10 US EDs in 2008-2009. The study questionnaire was conducted as the initial assessment of a prospective cohort study of smoking and novel predictors of smoking cessation among ED patients. During a 10-day enrollment period, trained research staff screened consecutive ED patients for tobacco use during peak volume hours (9:00 AM to midnight). Each site enrolled a minimum of 36 subjects.

All subjects received treatment-as-usual by ED providers for their tobacco use; however, research staff gave subjects an educational pamphlet on smoking cessation published by the US Department of Health and Human Services and a list of tobacco-cessation treatment options, which included the National Quitline number. Furthermore, subjects who screened positive for depression, alcohol, or drug use were given educational pamphlets published by the Association for Behavioral and Cognitive Therapies. In addition, subjects received brochures with national mental-health hotlines and state-based behavioral health referral services, which could be used to identify mental-health and substance-abuse treatment options.

The Institutional Review Boards of the participating institutions approved this study.

### Study setting and population

The 10 participating EDs (located in Phoenix, AZ; Loma Linda, CA; Denver, CO; Baton Rouge, LA; Camden, NJ; Bronx, NY; New York, NY; Akron, OH; Cleveland, OH; and Portland, OR) were selected for their diverse geographic, socioeconomic, and ethnic/racial patient populations. All were urban teaching-hospital EDs staffed by attending and resident physicians and physician extenders. The annual patient volume at these institutions ranged from 35,000 to 87,000. The majority serve a population that is > 25% African American, and four serve populations that are > 20% Hispanic. More than 20% of patients in most of the participating institutions lacked health insurance. No participating ED had formal tobacco intervention services or treatment protocols that would distinguish them from the typical US ED.

Patient inclusion criteria were age 18 years or older and daily or current occasional cigarette use. We excluded patients with altered mental status, acute intoxication, hostile or agitated behavior, an insurmountable language barrier, transient residence, lack of access to a telephone, or severe illness that would preclude conversation. Sites maintained a registry with all patients registered in the ED during the shift to facilitate a comparison of enrolled patients to those not enrolled.

### Data collection

Subjects completed a self-report, paper-and-pencil baseline assessment during their ED visit. The assessment included questions on smoking-related variables, predictors of cessation, and interest in a variety of hypothetical ED-initiated tobacco interventions and counseling styles. It took 15-20 min to complete. All measures were printed in both English and Spanish. The assessment was completed through research staff interview when necessary to accommodate patients with poor eyesight or illiteracy. This was done for < 5% of subjects. To reduce demand bias, which could lead to under-reporting of tobacco use and over-reporting of interest in cessation or interventions, participants were reassured that their responses would not be revealed to their treating clinicians. The specific variables we assessed are described under the Measures section.

In addition to the self-report measures, trained research staff completed a structured chart review for each subject, noting a variety of characteristics associated with the visit including the diagnoses assigned to the ED visit, triage level, disposition (admitted versus discharged), and documentation of smoking-related management, such as whether the ED clinicians provided smoking cessation counseling or referrals to the individual.

The study was coordinated by the Emergency Medicine Network (EMNet). Data collection forms were reviewed by EMNet staff, and missing or inconsistent data were reconciled through communication with the site. All data underwent double data entry.

### Measures

A broad range of measures were included in the parent study. The focus of this paper is on the interest in ED-initiated interventions and counseling styles. The specific measures used in the data analyses are described below.

### Demographics

Demographic characteristics included age, gender, race, ethnicity, education, insurance status and income.

### Tobacco use and nicotine dependence

Current smokers were defined as anyone who had smoked at least 100 cigarettes in their lifetime and reported currently smoking cigarettes every day or some days. Although most randomized trials of tobacco interventions only include daily smokers, including nondaily smokers helps account for the tendency of medically ill smokers to temporarily decrease or stop smoking because they are feeling poorly, not because they are actively trying to quit [21]. Nicotine dependence was assessed with the Heavy Smoking Index (HSI) [22,23], a well-established two-item self-report measure for use when rapid assessment is needed. Strength of nicotine dependence is represented by the sum of cigarettes smoked per day (0 = 1-10; 1 = 11-20; 2 = 21-30; 3 = 31+) and the time until first cigarette (0 = 61+ minutes; 1 = 31-60; 2 = 6-30; 3 = 0-5). Scores between zero and three indicate low to moderate dependence, and scores greater than three indicate high dependence. The HSI correlates highly with the Fagerström Test for Nicotine Dependence, the most widely use measure of nicotine dependence, and has been shown to be positively associated with carbon monoxide levels [24].

### Interest in ED-initiated interventions

We created a list of possible interventions that could be initiated in the ED and assessed patient interest using a 5-point Likert-type scale (1 = not at all interested, 5 = extremely interested). The interventions consisted of 1) reading a pamphlet or watching a video that describes ways to stop smoking, 2) receiving a list of telephone numbers for places to get stop-smoking counseling, 3) having one's name and telephone number sent confidentially to a stop-smoking counselor so he/she can call you at home to discuss treatment options, 4) having an actual appointment with a stop-smoking counselor scheduled within the next four weeks (before leaving the ED), 5) stop-smoking counseling during the current ED visit, 6) getting a prescription for NRT, such as the patch, gum, lozenge, or spray, upon discharge, 7) getting a prescription for medication that help people quit, like bupropion or varenicline, at discharge, and 8) being enrolled in an 8-week stop-smoking program. Individuals who expressed interest in ED counseling were asked how many minutes would be acceptable: 0-5 min, 6-15 min, 16-30 min, 30-45 min, or "no limit, as long as it doesn't delay my care."

### Counseling styles

We asked subjects to rate their acceptance of a variety of different counseling messages on a 5-point Likert type scale (1 = strongly dislike, 5 = strongly like). The counseling messages were 1) the counselor or doctor explains to you the health risks of smoking; 2) the counselor or doctor tells you that you should quit smoking immediately; 3) the counselor or doctor asks you some questions that help you to identify your own reasons for quitting, as well as barriers that prevent you from quitting; 4) the counselor or doctor explains that you are addicted to nicotine; 5) the counselor or doctor asks you some questions that help you identify high-risk situations and teaches you how to avoid smoking in these situations; 6) the counselor or doctor shows you pictures of people's lungs after they have smoked for years to try to scare you into quitting; 7) the counselor or doctor tells you that you should be ashamed of yourself for smoking; 8) the counselor or doctor explains that it is your own choice of when and how you quit; and 9) the counselor or doctor does an assessment and gives you feedback on how smoking has already affected your health. Some items were consistent with motivational interviewing (MI) principles, while others were not.

### Smoking-related symptoms

We assessed a list of smoking-related symptoms and diagnoses over the past 12 months using "yes/no" questions, including heart disease or heart attack, high blood pressure, stroke, problems with blood circulation, peripheral vascular disease, cancer, chronic obstructive pulmonary disease (COPD), emphysema, bronchitis, congestive heart failure, wheezing, shortness of breath, respiratory or sinus infection, cough, congestion, pneumonia, and asthma. A sum score was calculated representing the total number of symptoms and diagnoses endorsed.

### Smoking-related diagnoses

All *International Statistical Classification of Diseases and Related Health Problems *(9^th ^ed.) (ICD-9) diagnoses assigned by trained ED coders and used for billing purposes were obtained on all subjects. The diagnoses were categorized based on whether they met criteria for a smoking-related disease as outlined by the US Surgeon General. This is a commonly used strategy to classify smoking-related diseases and has been applied successfully to ED patients [25]. Two variables were created based on whether the primary ED diagnosis was smoking-related and whether any ED diagnosis was smoking-related.

### Emergency department evaluation and management

Trained research assistants (RAs) reviewed medical records of participants and abstracted relevant visit data using a standardized form. Charts were reviewed for clinical data, including triage level, patient disposition, and ICD-9 diagnoses assigned to the visit, and for documentation of patient tobacco use. If smoking was documented, RAs noted if clinicians indicated amount of tobacco use (packs per day or years) and if they provided counseling, smoking discharge instructions, NRT, or referrals to outpatient quitting resources.

### Data analysis

Descriptive statistics are presented as means with standard deviations or counts with percentages and 95% confidence intervals. For the primary objective of the study (to examine patient preferences regarding tobacco interventions), in addition to reporting the results based on a 5-point Likert scale, interest in each counseling type was converted to a dichotomous variable (interested/not interested). As participants rated preferences on a 5-point scale, with 3 as the "neutral" option, interest in an intervention was defined as a score greater than 3.

For the secondary research objective (to examine how patient characteristics affected preferences for receiving ED-based counseling for smoking cessation), we first examined variables likely to be influential on smoking based on the literature, including demographics (age, gender, race/ethnicity, education), amount of smoking (by HSI), smoking-related symptoms, and smoking-related diagnoses [26,27]. We assessed these variables individually for association with interest in ED counseling using t-tests or Pearson correlation as appropriate. Finally, we developed a linear regression model for interest in ED counseling, including variables that reached statistical significance by *p *< 0.10 in the univariable analysis. All analyses were performed using SPSS 17.0 (Chicago, IL, USA).

## Results

### Subject demographics

Of the 3662 patients considered for the study, 2132 (58%) were nonsmokers; 590 (16%) had a medical, psychological, or mental status problem preventing approach; 192 (5%) refused to be screened; 92 (3%) had an insurmountable language barrier; 106 (3%) were not able to be followed over time; and 172 (5%) were not enrolled for other reasons (primarily because they left without being seen or against medical advice, they were in police custody, or they were discharged prior to approach by research staff). Of the 378 patients originally enrolled in the study, three were removed because of missing data on more than five of 10 items considered critical for the study (e.g., smoking rate, stage of change, confidence in quitting), leaving 375 participants for analysis. Participant characteristics are shown in Table 1. Overall, participant demographics represented the diverse urban populations from which they were recruited: 37% were black, 20% were Hispanic/Latino, and 64% had annual household incomes of $40,000 or less. Compared with patients who were not approached or who refused to participate, subjects were more likely to be younger, to have Medicaid insurance, and to be discharged from the ED versus admitted (*p *< 0.05 for each characteristic). No differences were observed between those enrolled and those not enrolled in terms of sex or race. Forty-seven percent of participants smoked at least half a pack of cigarettes a day, and 80% smoked within 1 h of awakening in the morning.

**Table 1:** Patient Characteristics (N = 375)

Characteristic	n (%)
Age (mean)	41

Gender	
Female	210 (56)
Male	164 (44)

Race/ethnicity	
Hispanic/Latino	73 (20)
White, non-Hispanic	152 (41)
Black, non-Hispanic	137 (37)
Other	10 (3)

Insurance categories	
Private	79 (22)
Medicare	22 (6)
Medicaid or other public	129 (37)
Uninsured	123 (33)

Annual household income	
< 20,000	148 (40)
≤ 21,000-40,000	91 (24)
41,000-60,000	34 (9)
61,000-80,000	11 (3)
> 80,000	12 (3)
Don't know	79 (21)

Highest grade completed	
< high school	88 (24)
high school	282 (76)

Smoking measures:	

Cigarettes per day	
(1-10)	198 (54)
(11-20)	118 (32)
(21-30)	36 (10)
(≥ 31)	19 (5)

Minutes after waking to 1^st^	
cigarette	71 (20)
(> 61)	41 (11)
(31-61)	125 (34)
(6-30)	127 (35)
(0-5)	

### Preferences for tobacco cessation interventions

Patient preferences for ED-initiated interventions are shown in Table 2. Most participants (75%) reported interest in at least one of the listed interventions, although only one (a prescription for NRT) appealed to > 50% of smokers. Participants most often expressed interest in interventions that did not require considerable staff effort, such as receipt of a stop-smoking pamphlet (42%) or list of telephone numbers for smoking-cessation counseling (43%). Those interventions requiring more staff effort, such as counseling during the ED visit (33%) or faxed referral to outpatient counseling (37%), were slightly less popular. Of the individual counseling styles suggested (Table 3), those rated most favorably involved individualized feedback (54%), skill-building to avoid smoking (53%), and emphasis on autonomy (53%).

**Table 2:** Interest in ED-Initiated Tobacco Interventions (Listed in Order of Intensity)

Type of intervention	Likert scale score (%)					Any interest (> 3 on Likert scale) % (95% CI)
		
	1 (not at all interested)	2	3	4	5 (extremely interested)	
Stop smoking pamphlet or video	30	10	19	14	27	42 (37-47)

List of telephone numbers for stop-smoking counseling	28	13	16	12	31	43 (38-49)

Call from stop-smoking counselor	39	9	13	13	26	39 (34-44)

Appointment with stop-smoking counselor within next 4 weeks	33	10	13	14	29	43 (38-48)

Prescription for NRT	27	7	12	12	43	54 (49-59)

Prescription for (other) medication that helps people quit smoking	33	7	14	9	37	47 (41-52)

Stop-smoking counseling during ED visit	44	10	13	11	22	33 (28-38)

Being enrolled in an 8-week stop-smoking program	42	7	15	11	26	37 (32-42)

Interest in any intervention						75 (70-79)

**Table 3:** Interest in Counseling Styles

Counselor	Likert scale score (%)					Any interest (> 3 on Likert scale) % (95% CI)
		
	1 (not at all interested)	2	3	4	5 (extremely interested)	
Tells you that you should be ashamed of yourself for smoking	63	7	14	5	11	16 (12-20)

Shows pictures of people's lungs after they have smoked for years to try to scare you into quitting	35	9	21	11	25	36 (31-41)

Tells you that you should quit immediately	25	10	27	9	29	38(33-43)

Explains that you are addicted to nicotine	27	8	25	11	29	40(35-45)

Explains risks of smoking	24	9	27	10	31	41 (36-46)

Helps identify your reasons for quitting and barriers that prevent you from quitting	14	10	25	16	35	51 (46-56)

Helps identify high-risk situations and teaches you how to avoid smoking in these situations	16	6	26	17	36	53 (47-58)

Tells you that it is your choice of when and how you quit	18	6	23	14	39	53 (48-58)

Provides health assessment and feedback on how smoking has already affected your health	15	8	24	14	40	54 (49-59)

### Predictors of interest in ED-based tobacco cessation counseling

We also examined potential predictors of interest in ED bedside counseling. In univariable analysis (Table 4), age, gender, race, education level, and presence of smoking-related symptoms were significant and were included as covariables in the linear regression model. In the multivariable model (Table 4), male gender, failure to complete high school, and presence of self-reported smoking-related symptoms were all significant independent predictors of increased receptivity to ED bedside counseling. There were no gender-based differences in expressed interest in any other smoking intervention.

**Table 4:** Predictors of Interest in ED Bedside Counseling

	Univariable tests		Linear regression model
**Patient Characteristic**	**Likert Score, Mean (SE) or r (correlation)**	p*	**β coefficient (95% CI)**

Age	0.09	0.06	0.006 (-0.01, 0.02)

Gender		0.05	-0.45 (-0.80, -0.10)
Male (0)	2.75 (0.13)		
Female (1)	2.42 (0.11)		

Race/ethnicity		0.01	-0.40 (-0.75, -0.05)
Non-white (0)	2.75 (0.13)		
White (1)	2.31 (0.12)		

Insurance		1.00	
Insured	2.57 (0.14)		
Uninsured	2.57 (0.11)		

Education		0.03	-0.40 (-0.81, -0.01)
Finished high school	2.44 (0.10)		
Did not finish high school	2.92 (0.19)		

Smoking-related ICD-9 diagnosis		0.52	
Yes	2.73 (0.28)		
No	2.55 (0.09)		

Heavy Smoking Index (HSI)	0.03	0.60	

Smoking-related symptoms	0.10	0.05	0.09 (0.01, 0.16)

*Variables with *p *< 0.10 were included in the linear regression model.

In most cases, clinician evaluation of smoking habits did not appear to be followed by further management. Although clinicians noted tobacco use as a component of the social history in most patients (87%), only 15% mentioned smoking cessation in their discharge instructions, and only 18% had any information related to tobacco cessation referrals. Only 4% of patients received documented counseling, and less than 1% received prescriptions for NRT.

## Discussion

Although a few prior studies have examined the acceptability, feasibility, and effectiveness of ED smoking interventions [28,29], this is the first study to assess actual patient preferences from among a range of tobacco-related intervention types. Overall, smokers in our study expressed receptivity to therapies to help stop smoking, with 75% interested in some kind of intervention. However, within the group there was considerable variation in individual preference, with most of the individual strategies appealing to less than 50% of the sample. In general, interventions requiring little provider time or resources, such as smoking pamphlets or quit-line referrals, were more popular among patients than those requiring more provider time, such as immediate counseling at the bedside or a faxed referral to outpatient counseling. These interventions also required little time or investment by patients themselves, which may explain these findings.

Participants also indicated interest in a broad range of counseling styles. Only one style--making the patient feel ashamed about smoking--was unpopular; even directive messages ("counselor tells you that you should quit immediately") and scare tactics ("counselor shows pictures of people's lungs after they have smoked for years to try to scare you into quitting") were viewed positively by many patients. The most popular counseling styles were those that emphasized patient autonomy and provided individualized feedback and coping skills--in sum, counseling styles that are consistent with motivational interviewing. Among potential predictors of receptivity to counseling, experiencing health problems related to smoking was associated with interest in ED counseling, corroborating the idea of the ED visit as a "teachable moment" in which individuals are open to behavioral change.

In 2006, a task force convened by the American College of Emergency Physicians (ACEP) called upon emergency physicians to routinely assess patients' smoking status, offer brief quit advice, and refer patients to quit lines or other locally available cessation programs [30]. Our study suggests that this model is likely to appeal to many patients, but that some patients may be receptive to more than the basic "ask, advise, refer" model [31]. The broad range in preferences for interventions and counseling types we observed supports developing, testing, and making available a wide range of treatment options that can be tailored to individual patient needs and characteristics, as well as the demands and capacities of the clinical setting. Unfortunately, our study also confirmed that physicians currently continue to do little more than gather information about smoking habits and fail to counsel, refer, or prescribe medications, such as NRT, even though a significant proportion of smokers are receptive to these actions.

### Is the ED an appropriate site for delivering immediate and personalized treatment plans for smoking cessation?

Use of the ED for preventive health practices has many barriers, including time constraints, lack of knowledge of appropriate and effective interventions, concerns about offending and alienating patients, clinician doubts about their ability to impact patient health through smoking interventions, and attitudes about the appropriate role of the ED in preventive health activities [32]. Growing evidence, including the current study, may help alter attitudes about patient-level resistance to interventions. However, further work is needed to increase systems supports for screening and referrals, to identify effective and feasible ED-based interventions, and to train ED staff in smoking cessation measures to actualize the role of the ED as a place "uniquely suited" to help individuals quit tobacco use [30,31].

### Limitations

Our study had a number of limitations. The study sample was selected during peak ED visit hours only and may not be truly representative of individuals who present to the study hospitals. However, the participant demographics and smoking habits were consistent with the overall populations of the participating EDs as well as with the populations of prior ED studies in urban settings [2,33,34]. All participating EDs were in urban academic centers; thus, our study findings may not be generalizable to all EDs, particularly those in rural or remote settings. However, the study sites did otherwise represent a wide diversity of patient volumes, geographic locations, and patient populations.

Some demographic differences may exist between the ED patients who enrolled in the study and those who were not approached or refused to participate. Also, those who participated may represent a population that is more ready to discuss their smoking, the links between smoking and illness, and the means to address their smoking, than those who did not agree to participate. This may have led to an overestimation of patient receptivity to tobacco interventions.

In the questions about potential ED interventions, all questions used the phrase "stop smoking" or "helps to quit" except the one related to NRT, which did not refer specifically to cessation. This may have created a bias in which respondents were more likely to select the option that referred specifically to quitting smoking. On the other hand, NRT may have been more appealing because it was phrased in a way that implied (by not mentioning quitting) modification, rather than cessation, was possible. Nicotine replacement was the most popular option selected by study subjects; the effect of this question bias may have been to diminish or exaggerate its appeal to our patients.

Our assessment of management of tobacco use depended on the documentation practices of clinicians in the ED. It may be that tobacco counseling and referrals occurred but were not documented. However, the rates of counseling noted in our study were actually higher compared to prior research demonstrating low rates of ED-initiated tobacco counseling and referrals to treatment [10]. Our study likely presents a "best-case scenario," with enhanced tobacco-related counseling and referrals occurring because providers knew that a study was occurring in the ED, and/or because the study procedures prompted patients to speak about smoking with their clinicians more often.

## Conclusions

In this study, we found the majority of smokers in the ED are willing to engage in tobacco-related interventions. Patients expressed interest in a wide variety of interventions and counseling types, while clinicians often noted tobacco use but rarely followed through with treatments or referrals. Clinicians should not assume smokers in the ED are resistant to receiving health messages and counseling related to their tobacco use. Further, researchers may consider developing a range of options for assisting individuals in the ED who are interested in reducing their tobacco use.

## Competing interests

The authors declare that they have no competing interests.

## Authors' contributions

Author EDB designed the study and wrote the protocol. Author EKC performed literature searches, reviewed the statistical analysis, wrote the first draft of the manuscript, and was responsible for subsequent manuscript revisions. Author EDB performed the statistical analysis. All authors (EKC, AFS, FL, JNP, CAC, EDB) participated in acquiring the data, analyzing and interpreting the data, and performing critical revision of the manuscript content. All authors approved the final manuscript.
